# Brucellosis Presenting as Isolated Abdominal Lymphadenopathy Masquerading as Lymphoma: A Case Report

**DOI:** 10.7759/cureus.56325

**Published:** 2024-03-17

**Authors:** Nicolas Sandakly, Georgio El Koubayati, Samah Naderi, Delivrance Sebaaly, Fady Haddad

**Affiliations:** 1 Infectious Diseases, Internal Medicine, Lebanese University Faculty of Medicine, Beirut, LBN; 2 Immunology, Lebanese University Faculty of Medicine, Beirut, LBN; 3 Pathology, Lebanese Hospital Geitaoui, Beirut, LBN; 4 Internal Medicine and Clinical Immunology, Saint Joseph University, Faculty of Medicine, Beirut, LBN

**Keywords:** lymphoma, abdominal lymphadenopathy, brucellosis

## Abstract

Mesenteric lymphadenopathy associated with high-grade fever can be frequently associated with hematologic malignancies, especially if accompanied by joint pain, weight loss, and anorexia. However, this constellation of symptoms, also known as "B Symptoms," can be the uncommon presenting manifestation of brucellosis, still a common zoonotic disease in the Middle Eastern basin. In this article, we report the case of a Lebanese man who presented with “B symptoms” of three weeks duration, who was thought to have lymphoma but was later found to have systemic brucellosis.

## Introduction

Brucellosis, commonly referred to as undulant or Malta fever, is still one of the most common zoonoses worldwide. The infection is caused by an intracellular gram-negative, non-spore-forming *Coccobacilli* bacteria of different species, with *Brucella melitensis* being the most pathogenic for humans. It is endemic in the Mediterranean basin and the Middle East [1] and is mainly contracted by the ingestion of unpasteurized milk, dairy products, or undercooked meat from infected reservoirs [2]. The clinical manifestations of brucellosis can range from asymptomatic to multi-organ involvement, with fever, drenching night sweats, joint pain, and lymphadenopathies being the most commonly reported symptoms [3], and pancytopenia and lymphocytosis being the most commonly encountered laboratory anomalies [4]. These manifestations can also be the patterns of presentation of lymphoproliferative disorders, including lymphomas, and are known as ‘B symptoms. As a matter of fact, they are seen in 68% of patients with stage IV Hodgkin’s lymphoma and in 10% of patients with Hodgkin’s lymphoma [5,6]. However, isolated lymphadenopathy is rarely due to malignancy, with one study reporting the prevalence of malignancy to be as low as 1% in primary care patients presenting with unexplained lymphadenopathies [7]. In the following article, we report the case of a man admitted for ‘B symptoms’ and lymphadenopathy, whose initial work-up was suggestive of lymphoma but was eventually diagnosed with brucellosis.

## Case presentation

A 48-year-old male with no significant medical history was admitted to the Lebanese Hospital Geitaoui University Medical Center (LHG-UMC), presenting with a three-week history of fever, night sweats, abdominal pain, and back pain.

He is a carpenter. He denied exposure to raw animal fluids or meat, has no pets at home, and is in a monogamous heterosexual relationship. The patient reported a 7 kg (15.43 lb.) weight loss during this period. He denied any history of trauma or injury, recent travel, or contact with sick individuals. Upon admission, vital signs were stable except for a temperature of 38.5°C. A physical examination was relevant for epigastric and left upper quadrant tenderness. There are no palpable cervical, axillary, or inguinal lymphadenopathies. Initial laboratory studies (Table 1) showed pancytopenia with a hemoglobin level of 11.9 g/dl, white blood cells at 3010 cells/mm3, and platelet count at 110,000/mm3. LDH was elevated at 475 U/L. Liver and kidney function tests were normal. C-reactive protein (CRP) was elevated at 57 mg/L. ﻿

**Table 1:** Laboratory results for the patient. Laboratory results of the patient showed pancytopenia with high LDH and CRP. The Wright test was negative, but brucella-blocking antibodies came back elevated.

Laboratory exam	Patient’s result	Reference range (unit)
Hemoglobin	11.9	14-18 (g/dL)
White blood cells	3,010	4,800-10,800 (cells/mm3)
Platelets	110,000	130,000-400,000 (/mm3)
Lactate Dehydrogenase (LDH)	475	91-248 (u/L)
C-reactive protein (CRP)	57	<6 (mg/L)
VCA IgM anti-EBV	5	<20 (U/mL)
IgM anti- CMV	3.2	<20 (U/mL)
Wright test	<1/20	<1/20
Brucella Blocking Antibodies	1/10240	<1/20
Antinuclear Antibodies (ANA)	<1/20	<1/40

**Figure 1:**
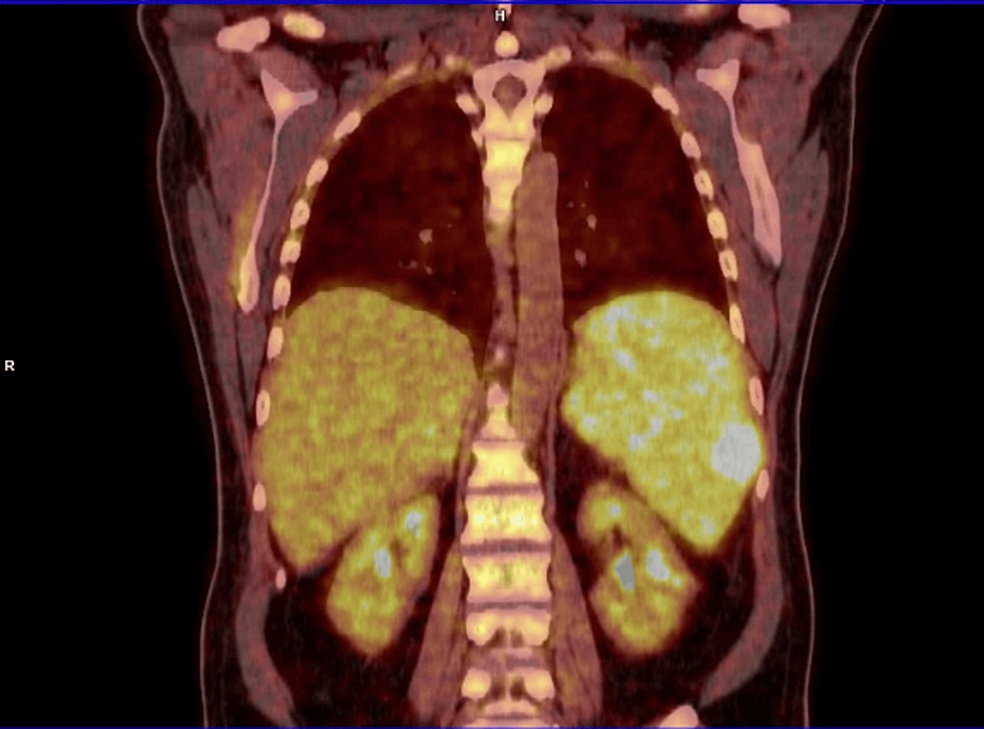
Illustration of abnormal diffuse and focal spleen FDG radiotracer uptake and homogenous hepatomegaly with physiologic FDG radiotracer uptakeen PET-CT of the patient shows abnormal diffuse and focal spleen FDG radiotracer uptake and homogenous hepatomegaly with physiologic FDG radiotracer uptakeen

Urine analysis yielded negative results. Blood cultures were obtained, and the patient was started on ceftriaxone. A contrast-enhanced abdominal and pelvic computed tomography (CT) scan revealed mild hepatomegaly of 18.5cm and a moderate splenomegaly of 17cm. Numerous mesenteric and retroperitoneal lymph nodes were also noted. A positron emission tomography (PET) scan (Figure 1) was performed the next day, and findings of hepatosplenomegaly and hyperavid lymph nodes were suggestive of lymphoma and were biopsied for diagnostic purposes. 

**Figure 2:**
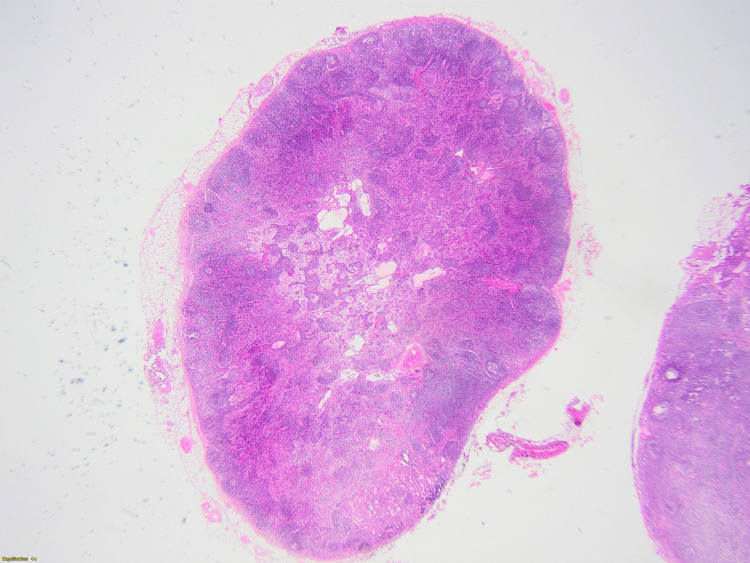
Histopathological findings of the mesenteric lymph node Preserved architecture (hematoxylin and eosin ×40)

**Figure 3:**
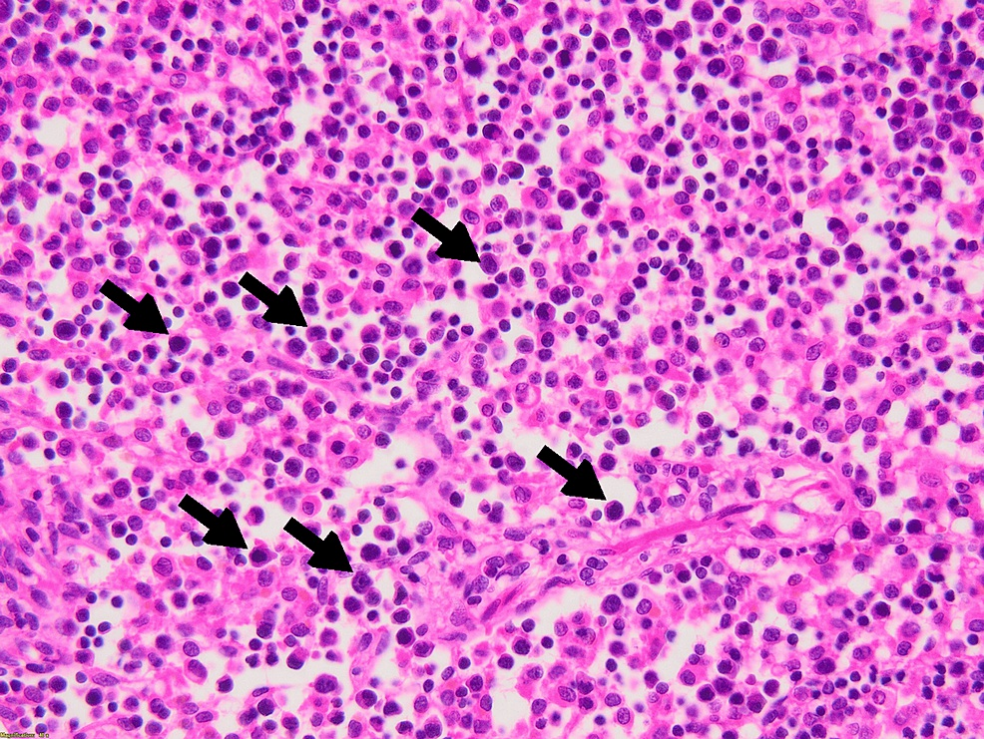
Positive expression for CD30 and CD43 without evidence of expression of ALk D5F3 and CD15 on immunostaining The sinusoids are populated by atypically medium-sized cells with a high nucleocytoplasmic ratio and irregular nuclei. Multiple mitoses are noted (black arrows).

**Figure 4:**
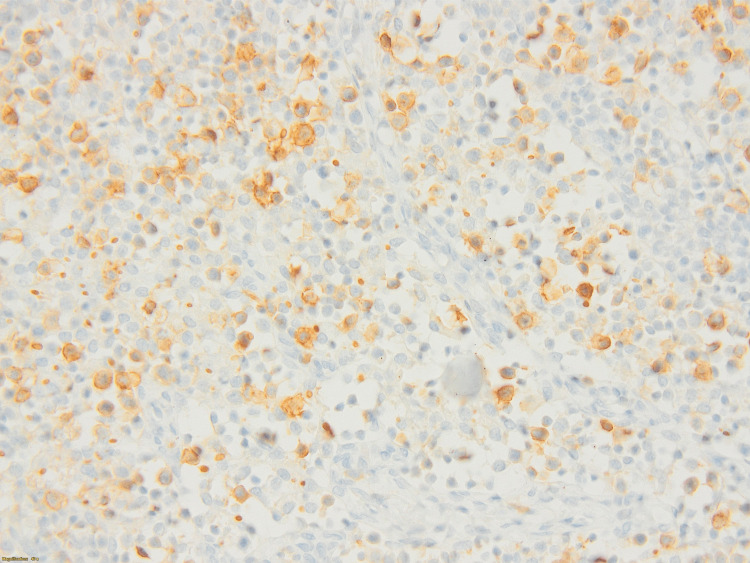
Intrasinusoidal, atypical medium-sized cells with a high nucleocytoplasmic ratio and irregular nuclei (hematoxylin and eosin ×400) The immunostaining reveals that these cells are positive for CD30 and CD43, without evidence of expression of ALK D5F3 and CD15.

On the third day of admission, the patient became neutropenic with an absolute neutrophil count (ANC) of 1012, requiring placement under reverse isolation. Autoimmune workup and a purified protein derivative (PPD) skin test for tuberculosis came back negative. The EBV and CMV serologies yielded negative results. The Wright agglutination test for brucellosis came back negative, although a thorough history of the patient revealed recent consumption of unpasteurized dairy products. Biopsy results raised the hypothesis of an anaplastic T cell lymphoma with atypical medium-sized cells positive for CD30 and CD43, with a high nucleocytoplasmic ratio and irregular nuclei, without expression of ALk D5F3 and CD15 on immunostaining (Figures 2-4). 

On the sixth day of admission, *Brucella melitensis* was isolated from the blood culture, and brucella-blocking antibodies were positive at 1/10240. In light of these findings, a diagnosis of brucellosis was established, further supported by positive serology performed for his brother, who presented to our outpatient clinic for non-specific abdominal pain and also reported consuming unpasteurized dairy products. The patient was started on intravenous gentamicin (5 mg/kg/day), rifampin 300 mg twice daily PO, and doxycycline 100 mg twice daily PO. The fever subsided on the third day of antibiotic initiation, and the patient reported clinical improvement. The patient recovered from his neutropenia on the fifth day after antiotherapy and was discharged home after 14 days on rifampin and doxycycline for six weeks. The treatment protocol was well tolerated by the patient, and a metabolic panel (including a complete blood count and liver and renal function tests performed every two weeks during treatment and one month after finishing antibiotherapy) was within reference range. The patient reported complete resolution of his symptoms at six-month follow-up, with a normalized cell count and absent malignant cells or blasts on peripheral smears. Injected CT scans of the abdomen and pelvis at six-month and one-year follow-up showed complete resolution of the lymphadenopathy and hepatosplenomegaly.

## Discussion

*Brucella* infection continues to present a substantial challenge, particularly in low- and middle-income countries, most notably in the Mediterranean rim and the Middle East. Notably, Lebanon has been a focal point in one of the most significant global outbreaks of brucellosis. In the years 2017 and 2018, a total of 1,180 cases were reported, marking the fifth documented outbreak of brucellosis since the last one in Algeria in 2016 [8]. Brucellosis can present with a wide array of non-specific signs and symptoms, overlapping with a variety of infectious and non-infectious diseases. Patients most frequently present with fever, fatigue, arthralgia, myalgia, and gastrointestinal symptoms [9]. Moreover, it can manifest in lymphadenopathies. In a retrospective study involving 1,028 cases of brucellosis, lymphadenopathy was present in 2.4%, with the highest being reported in acute brucellosis [10]. Furthermore, a systematic review and meta-analysis study including 68 publications related to the clinical presentation of human brucellosis with 12,842 patients from China reported lymph node involvement in up to 19% [11]. However, isolated localized adenopathy is a rare presentation of *Brucella* infection and has been limited to case reports. Varona et al. [12] documented a case of *Brucella* infection presenting as an isolated cervical lymphadenopathy. Massoud et al. [13] and Mirijello et al. [14] reported two case reports of isolated abdominal lymphadenopathy associated with brucellosis, to which we add this case report of a patient who presented with non-specific symptoms and mesenteric and retroperitoneal lymphadenopathy, a clinical presentation that was highly suggestive of lymphoma and found to have brucellosis. The diagnosis of brucellosis necessitates either the isolation of *Brucella* from the blood or body tissues or the combination of serology and clinical manifestations suggestive of the disease. Cultural examinations are time-consuming, and the detection of high or raised titers of specific antibodies in the serum allows for a tentative diagnosis. *Brucella* indirect Coomb’s test is more sensitive and specific than the standard agglutination titer (SAT), and when applied with the SAT, false negative results are avoided [15]. Using at least two serological tests increases the chances of a diagnosis of brucellosis [16]. In our case report, the positive indirect Coomb’s test result and the presence of risk factors for brucellosis, that is, the consumption of unpasteurized dairy products in a region known to be endemic for *Brucella* infection, further supported the diagnosis of brucellosis in our patient, which was confirmed by the isolation of *Brucella* species from blood culture, ruling out other differential diagnoses, including lymphoma, which was suggested by the PET scan and lymph node biopsy results, and considered that of atypical lymphoid hyperplasia within the context of brucellosis. The diagnosis was corroborated by the complete resolution of lymphadenopathy and hepatosplenomegaly after completing the treatment protocol at six-month and one-year follow-up. In fact, PET/CT scans show moderate sensitivity and low specificity in the diagnosis of lymphoma in patients presenting with fever accompanied by lymphadenopathy. In a prospective study including 162 patients with fever and adenopathy, PET/CT scans correctly diagnosed 104 cases when compared with the pathological results [17]. Inflammatory and infectious processes, in particular granulomatous diseases such as brucellosis, tuberculosis, aspergillosis, etc., can produce false positive results, in particular during the fulminant process. On the other hand, neoplasms with lower metabolic activities can give borderline or even false negative results [18]. Physicians should have a high index of suspicion for *Brucella* infection, especially in *Brucella*-endemic areas, when dealing with patients presenting with fever and lymphadenopathy. Beyond the diagnostic dilemma, the case discussed in this study underscores the significance of a comprehensive and thorough clinical history and the appropriate interpretation of the laboratory, radiological, and histological findings to accurately reach a diagnosis and establish the optimal treatment plan. 

## Conclusions

Brucellosis, which presents as isolated mesenteric lymphadenopathies, is infrequently reported. It is crucial to acknowledge that brucellosis can mimic lymphoma on PET scans and biopsy results. The case emphasizes the importance of considering brucellosis in the differential diagnosis of patients with fever and lymphadenopathies, particularly in endemic regions, and underscores the necessity of accurate interpretation of paraclinical tests to avoid misdiagnosis and ensure optimal patient care.
